# Olive Oil Polyphenols Improve HDL Cholesterol and Promote Maintenance of Lipid Metabolism: A Systematic Review and Meta-Analysis of Randomized Controlled Trials

**DOI:** 10.3390/metabo13121187

**Published:** 2023-12-06

**Authors:** Roberta Zupo, Fabio Castellana, Pasquale Crupi, Addolorata Desantis, Mariangela Rondanelli, Filomena Corbo, Maria Lisa Clodoveo

**Affiliations:** 1 Department of Interdisciplinary Medicine (DIM), University of Bari Aldo Moro, Piazza Giulio Cesare 11, 70100 Bari, Italy; castellanafabio@gmail.com (F.C.); pasquale.crupi@uniba.it (P.C.); marialisa.clodoveo@uniba.it (M.L.C.); 2 Department of Pharmacy-Drug Sciences, University of Bari “Aldo Moro”, 70125 Bari, Italy; addolorata.desantis@uniba.it (A.D.); filomena.corbo@uniba.it (F.C.); 3 Department of Public Health, Experimental and Forensic Medicine, University of Pavia, 27100 Pavia, Italy; mariangela.rondanelli@unipv.it

**Keywords:** olive oil polyphenols, HDL cholesterol, lipid metabolism, health claim, EFSA, randomized controlled trials, systematic review, meta-analysis

## Abstract

In 2011, the European Food Safety Authority (EFSA) accorded a health claim to olive oil polyphenols in that they protected LDL particles from oxidative damage. However, limited scientific evidence has so far failed to confer any claim of function on the maintenance of normal lipid metabolism. We performed a systematic review and meta-analysis of human RCTs, evaluating the effect of olive oil polyphenol administration on lipid profiles. Previous literature was acquired from six electronic databases until June 2023. A total of 75 articles were retrieved and screened for inclusion criteria, which resulted in the selection of 10 RCTs that evaluated the effect of daily exposure to olive oil polyphenols on serum lipids in adults. Meta-analyses were built by tertiles of outcomes, as follows: low (0–68 mg/kg), medium (68–320 mg/kg), and high (320–600 mg/kg) polyphenols for HDL and LDL cholesterol (HDL-C and LDL-C, respectively), and low (0–59.3 mg/kg), medium (59.3–268 mg/kg), and high (268–600 mg/kg) polyphenols for total cholesterol (TC). The study protocol was registered on PROSPERO (registration code: CRD42023403383). The study design was predominantly cross-over (*n* = 8 of 10) but also included parallel (*n* = 2 of 10). The study population was predominantly European and healthy. Daily consumption of olive oil polyphenols did not affect TC levels and only slightly significantly reduced LDL-C, with WMD statistically significant only for high daily consumption of olive oil polyphenols (WMD −4.28, 95%CI −5.78 to −2.77). Instead, our data found a statistically significant HDL-C enhancing effect (WMD pooled effect model: 1.13, 95%CI 0.45; 1.80, heterogeneity 38%, *p* = 0.04) with WMD by daily exposure level showing a statistically significant improvement effect for low (WMD 0.66, 95%CI 0.10–1.23), medium (WMD 1.36, 95%CI 0.76–1.95), and high (WMD 1.13, 95%CI 0.45–1.80) olive oil polyphenol consumptions. Olive oil polyphenols contribute toward maintaining lipid metabolism. Thus, food labeling regulations should stress this health feature of olive oil, whereby a declaration of the olive oil polyphenol content should be added to products on the market. Consumers need to be aware of the quality and possible health effects of any products they consume, and enforcement of nutrition labels offers the best way of providing this information.

## 1. Introduction

Cardiovascular disease (CVD) poses a major public health challenge and is a leading cause of death globally. In 2021, 17.9 million deaths, or 33% of all global deaths, were attributed to CVD, including 7.4 million due to coronary heart disease (CHD) and 6.7 million from stroke [1]. Most CVDs may be preventable by modifying lifestyle factors, such as unhealthy eating, physical inactivity, and tobacco use. Such behaviors result in risk factors for CVD that include increased blood lipids, blood pressure, blood sugar, and obesity. To some degree, rapid urbanization and changing lifestyles worldwide have driven increased consumption of foods high in saturated fats, sugars, and salt and lower intakes of fruits, vegetables, whole grains, and dietary fiber. Such dietary patterns have been linked to an Increased risk of CVD, while a prudent Mediterranean model protects against CVD [2]. Evidence has consistently shown that dietary factors [3] that reduce CVD risks are minimally processed foods, such as fruits, vegetables, nuts/seeds, beans/legumes, whole grains, and fish, although they also include a set of side metabolites, such as polyphenols, which possess antioxidant and anti-inflammatory properties [4].

Given this background, health claims on foods are an effective tool to communicate the relationship between the food and its specific health benefits to help consumers make healthy dietary choices. Upon the request of the European Commission, a panel was commissioned on Dietetic Products, Nutrition, and Allergies (NDA) to provide a scientific opinion on the substantiation of food health claims, to support consumer decision-making choices in a more informed, sustainable, and health-conscious direction [5]. Under Article 13 of Regulation (EC) No. 1924/2006, function claims refer to the effects that the food has on normal bodily functions based on the role that a food or its component plays when consumed at levels consistent with normal dietary patterns. Here, maintaining normal HDL cholesterol (HDL-C) concentrations (without increasing LDL cholesterol (LDL-C) concentrations) is considered a beneficial physiological effect. HDL-C is well known to play an anti-atherosclerotic role by protecting against the development of CVD; indeed, increased serum concentrations of HDL-C are strongly inversely related to the risk of atherosclerotic CVD [6,7].

Since modifiable lifestyle factors prevent cardiovascular risk, the Mediterranean diet has drawn extensive attention over recent decades for its beneficial effects on cardiometabolic and cardiovascular health [3,8,9]. A variety of compounds that have been attributed to this biological track include the polyphenolic content derived from vegetables, fruits, and olive oil. Yet, olive oil, particularly extra virgin olive oil (EVOO), has always been recognized as a symbol of the Mediterranean diet. A high consumption of EVOO, ranging from 15.3 to 23 kg per capita/year, is a staple of the Mediterranean diet and is one of the main differences from other healthy diets [10,11]. Historically, the olive tree (otherwise known as *Olea europea* L.) is a well-known evergreen tree in the Mediterranean basin, which is characterized by slow growth and an extremely long life expectancy, of up to 1000 years [9,10]. This species is one of the most important trees for the Mediterranean economy, providing many commercial products, such as food, timber, and cosmetics. However, oil remains the most important product provided by *Olea Europea* L. [9,11]. 

To date, evidence indicates that the beneficial impacts of EVOO occur because of its specific components. The high content of monosaturated fatty acids (C18:1, 55 to 83% of total fatty acids) is widely associated with the nutritional and health properties of this food [12]. However, interestingly, researchers have mainly focused on the minor component of olive oil (2% of total weight), which is rich in bioactive molecules [10,13]. Of these, phenolic compounds have been characterized by a broad spectrum of biological activities ranging from stability to auto-oxidation to beneficial effects on human health [14]. They mostly include glycides (e.g., oleuropein), alcohols, and phenols (tyrosol, hydroxytyrosol), yet also include flavonoids. Phenolic compounds are mainly responsible for the characteristic taste property of virgin olive oil, namely, the bitter taste. Health properties have been mainly attributed to their antioxidant, anti-inflammatory, cardioprotective, neuroprotective, anticancer, antidiabetic, and antimicrobial potentials [15,16,17,18,19]. 

Emerging preclinical and observational evidence has so far suggested that dietary polyphenol intake may reduce inflammation and also be associated with a reduction in all-cause mortality [20]. Virgin olive oil, compared to other dietary fats [21], carries a unique composition of polyphenols and is acknowledged as a cornerstone food in the Mediterranean model [22]. In particular, it contains a high concentration of hydroxytyrosol and oleuropein polyphenols, which have demonstrated cardioprotective properties in preclinical studies, including favorable modulation of pathways related to inflammation, oxidative stress, homocysteine, cholesterol levels, and cell adhesion [23,24,25].

In 2012, an attempt was made to substantiate a health claim related to polyphenols in olive oil and their maintenance of normal blood HDL-C levels (ID 1639) [26] based on a single RCT report. However, this was rejected by the European Food Safety Authority (EFSA) Panel owing to insufficient evidence of an established biological relationship between the consumption of olive oil polyphenols (standardized by the contents of hydroxytyrosol and its derivatives) and the maintenance of normal blood HDL-C levels. Nevertheless, a more substantial body of evidence from human randomized clinical trials (RCTs) on this topic may provide scientific robustness of a biological relationship between olive oil polyphenol consumption and the maintenance of optimal HDL-C levels; thus, possibly supporting a functional health claim.

We conducted a systematic review with a meta-analysis of randomized intervention studies to evaluate the efficacy of olive oil polyphenol consumption on HDL-C levels in humans, including a comprehensive appraisal of lipid biomarkers.

## 2. Methods

### 2.1. Search Strategy, Study Selection, and Data Extraction

No prior systematic reviews were found on the association between olive oil polyphenol exposure and serum HDL concentrations after performing a computerized literature search of the MEDLINE and Cochrane databases. The present systematic review followed the Preferred Reporting Items for Systematic Reviews and Meta-Analyses (PRISMA) guidelines, adhering to the PRISMA 27-item checklist [27]. Priori protocols for the search strategy and inclusion criteria were established and recorded, with no changes to the information provided at registration on PROSPERO, a prospective international registry of systematic reviews (CRD42023403383). We performed separate searches in the U.S. National Library of Medicine (PubMed), Medical Literature Analysis and Retrieval System Online (MEDLINE), EMBASE, Scopus, Ovid, and Google Scholar databases to find RCTs evaluating the effect of olive oil polyphenol intervention on lipid profiles in humans. Therefore, the primary objective was to evaluate whether the administration of olive oil polyphenols could benefit HDL-C concentrations without changing serum LDL-C or total cholesterol (TC) levels for the worse. We also considered gray literature using the huge archive of preprints https://arxiv.org/ (accessed on 15 June 2023) in the study selection phase and the database http://www.opengrey.eu/ (accessed on 15 June 2023) to access abstracts of notable conferences and other unreviewed material. 

The following criteria were applied to include specific studies in the analysis: (1) Randomized controlled clinical trials in humans with a crossover or parallel design; (2) reports the effects of olive oil polyphenols on at least three lipid profile parameters (HDL-C, LDL-C, TC); (3) studies that involved adult participants (over 18 years old); (4) intake of at least 20 g/day of olive oil. Animal studies, conference abstracts, nonclinical trial studies, and studies involving children and/or adolescents were not included. In addition, studies that used olive oil in combination with other oils or interventions, such as EPA and DHA or olive oil enriched with plant sterols were also excluded. 

The search strategy used in PubMed and MEDLINE and adapted to the other four electronic sources included keywords such as “olive oil”, “polyphenols”, and “lipid” combined using Boolean indicators such as AND and OR, as follows: “(olive oil) AND (polyphenol*) AND (lipid* OR cholesterol OR hdl OR ldl) NOT (review) NOT (letter) NOT (opinion) NOT (meta-analysis) NOT (editorial)”. The search strategy used the Boolean indicator NOT to exclude opinion papers, letters, reviews, and meta-analyses. The literature search had no time restrictions, and papers were retrieved until 1 July 2023. Two researchers (RZ, FC) searched the papers, reviewed the titles and abstracts of the retrieved articles separately and in duplicate, checked the full texts, and selected the articles for inclusion in the study. Inter-rater reliability (IRR) was used to estimate inter-coder agreement, and then the κ statistic to measure accuracy and precision. According to PRISMA concepts and the quality assessment steps, a coefficient k of at least 0.9 was obtained in all data extraction steps [28].

### 2.2. Quality Assessment

Two reviewers (RZ, FC) independently assessed the methodological quality of the eligible RCTs using the revised Cochrane risk-of-bias tool for randomized trials (RoB-2), which consists of five main domains, including bias arising from the randomization process, bias due to deviations from the intended interventions, bias due to missing outcome data, bias in the outcome measures, and bias related to the selection of reported results. Final judgments and overall risk of bias were defined as “low” or “high” risk of bias or expressed as “some concerns” [29]. A third reviewer (MLC) independently checked this assessment.

### 2.3. Data Analysis

Two researchers (RZ, FC) extracted and tabulated the following information separately and in duplicate in a piloted form: author, year of publication, country, population, design, inclusion and exclusion criteria, wash-out period, sample size, age, level of daily exposure to olive oil polyphenols (mg/Kg) (grouped as low, medium, and high consumption), and intervention duration (weeks or days). All references selected for retrieval from the databases were managed with the MS Excel data collection software platform by a senior biostatistician (FC). The data were extracted from the selected studies and stored in the database in structured evidence tables. Statistical analyses were conducted using the meta-package (R software, version 223.03.1). Multiple subgroup meta-analyses were performed to assess dietary exposure levels (categorized as low, medium, and high) of olive oil polyphenols on lipid variables of interest, i.e., HDL-C, LDL-C, and TC. Daily exposure to olive oil polyphenols was studied by tertiles of outcomes, as follows: low (0–68 mg/kg), medium (68–320 mg/kg), and high (320–600 mg/kg) polyphenols for HDL-C and LDL-C, and low (0–59.3 mg/kg), medium (59.3–268 mg/kg), and high (268–600 mg/kg) polyphenols for TC. 

Weighted mean differences (WMDs) with 95% confidence intervals (CIs) for HDL-C, LDL-C, and TC levels were calculated separately and then grouped by daily exposure in the olive oil polyphenols group in the meta-analysis. Mean changes in blood lipids were calculated by subtracting end-of-study values from baseline values. To avoid data duplications, only endpoint values were used for studies with more than one measure. If the SD of the mean change values was not available, the SD was calculated based on the 95% CI, otherwise, this measure was estimated from the standard deviations of the two assessment times using the following formula:

$$\mathrm{SD}\;\mathrm{change}=\sqrt{\mathrm{SD}2\;\mathrm{baseline}+\mathrm{SD}2\;\mathrm{final}-(2\;\times \;0.7\;\times \;SD\;\mathrm{baseline}\;\times \;SD\;\mathrm{final})}$$

where a correlation coefficient of 0.7 was adopted [30]. Blood lipid levels were collected in mg/dL, with the exposure to olive oil polyphenols in mg/kg. Thus, blood lipid values reported in mmol/L were converted to mg/dL. Heterogeneity was assessed by Cochran’s Q statistic and quantified (*I*^2^). The *I*^2^ statistic and P value were used to analyze heterogeneity among studies. A *p*-value < 0.1 or *I*^2^ > 50 was considered as meaningful heterogeneity between studies. The common effect model was applied for all analyses. All analyses were performed with R software version 223.03.1 by a senior biostatistician (FC). 

## 3. Results

**Figure 1:**
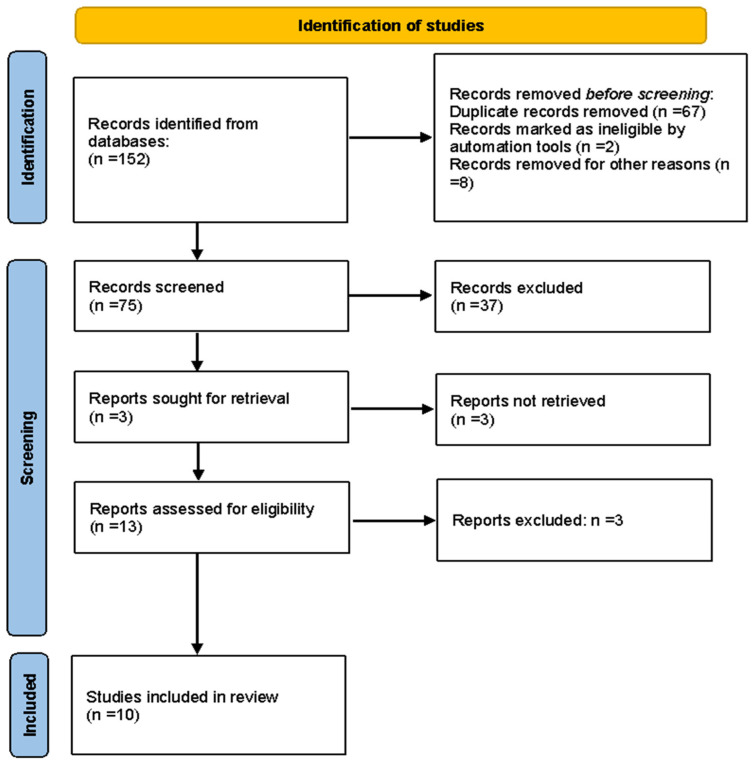
Flow chart depicting the screening process.

The first systematic search of the literature yielded 152 entries. After excluding duplicates, 75 were classified as potentially relevant and selected for the title and abstract analysis. Then, 37 were excluded for not meeting the characteristics of the approach or the review goal. After reviewing the full text of the remaining records, only 10 met the inclusion criteria and were included in the meta-analysis [31,32,33,34,35,36,37,38,39,40]. The Preferred Reporting Items for Systematic Reviews and Meta-analyses (PRISMA) flow chart illustrating the number of studies at each stage of the review is shown in Figure 1. The final study base included 10 RCT articles, which reported on the effect of olive oil polyphenol intervention on lipid profiles in humans.

Details of the design (cross-over or parallel-arm RCT, blinded or non-blinded), sample size (*n*), country, author(s), year of publication, population, inclusion and exclusion criteria, intervention duration and washout period, and daily olive oil polyphenol exposure levels (mg/kg) are provided in Table 1.

The crossover (80%, *n* = 8) prevailed over the parallel-arm design (20%, *n* = 2). Recruitment settings were all community-based, and the geographic distribution of studies favored Europe (80%, *n* = 8), with an Asian (10%, *n* = 1) and Australian minority (10%, *n* = 1) [32]. Only 4 of the 10 selected studies performed the dietary intervention in a double-blind way [31,33,35,40]. All subjects were healthy and adults (>18 years of age), except for two studies, which reported on type 2 diabetes mellitus [34] and hypercholesterolemic [35] subjects. Intake of antioxidant supplements, aspirin, or any other drug with established antioxidant properties was noted as an exclusion criterion in all selected studies. 

The age of the overall population ranged from 20 to 75 years old. A randomization methodology was used for all studies and employed a computerized random number generator, using Excel software. The intervention duration was three weeks for most studies (*n* = 6 of 10), with four weeks being used in two studies [34,39], three months in one study [37], and four days in another [39,40]. The treatment duration was not found to be statistically different among the studies, meaning it was not deemed necessary to structure the analyses based on the intervention duration.

The exposure levels were used as comparators between studies, with two found as a majority in six studies (low or high daily intake of olive oil polyphenols) [31,32,33,34,35,36] and three as a minority in four studies (low, medium, or high daily intake of olive oil polyphenols) [37,38,39,40]. The washout window was set at two weeks for six [31,33,35,36,38,39] of the eight crossover RCTs, otherwise, 4 weeks [34] or 10 days [40] were established for the remaining two studies. No washout period was applied to the two studies that used the parallel-arm RCT design [32,40].

**Figure 2:**
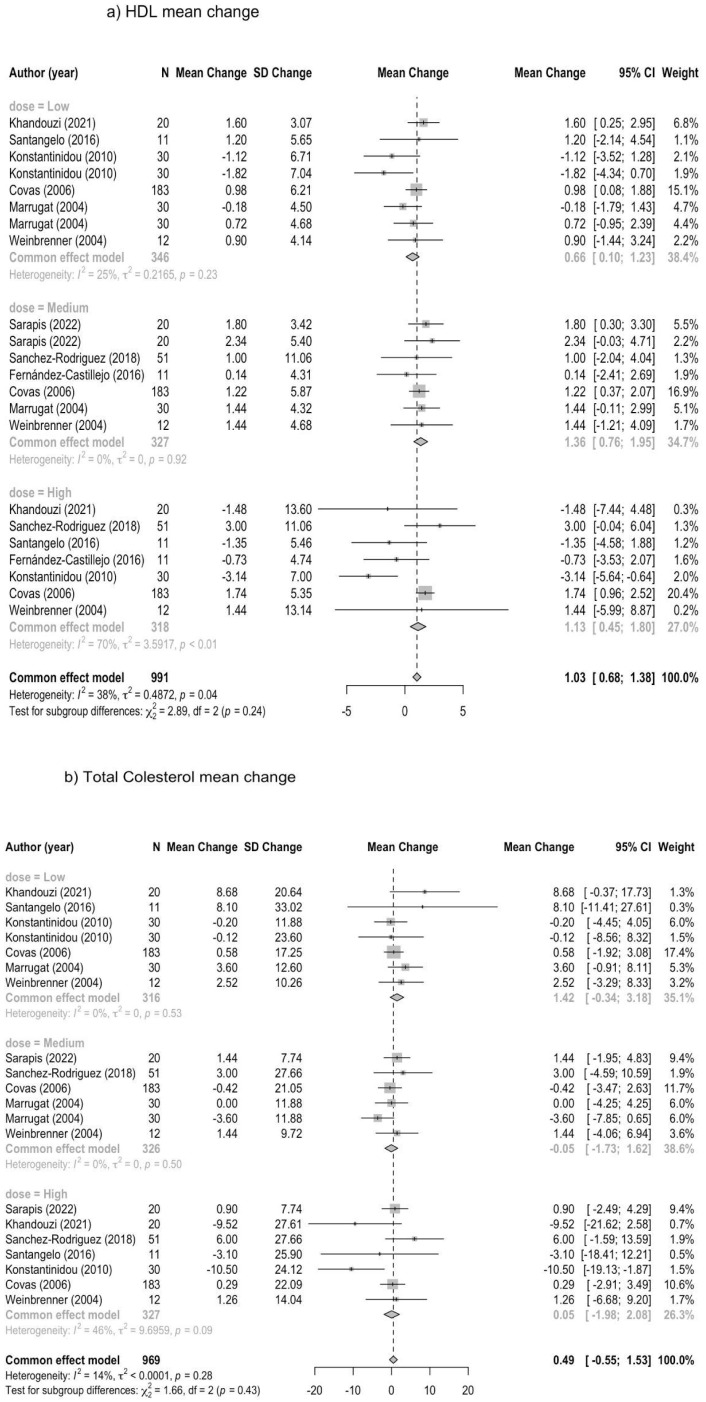

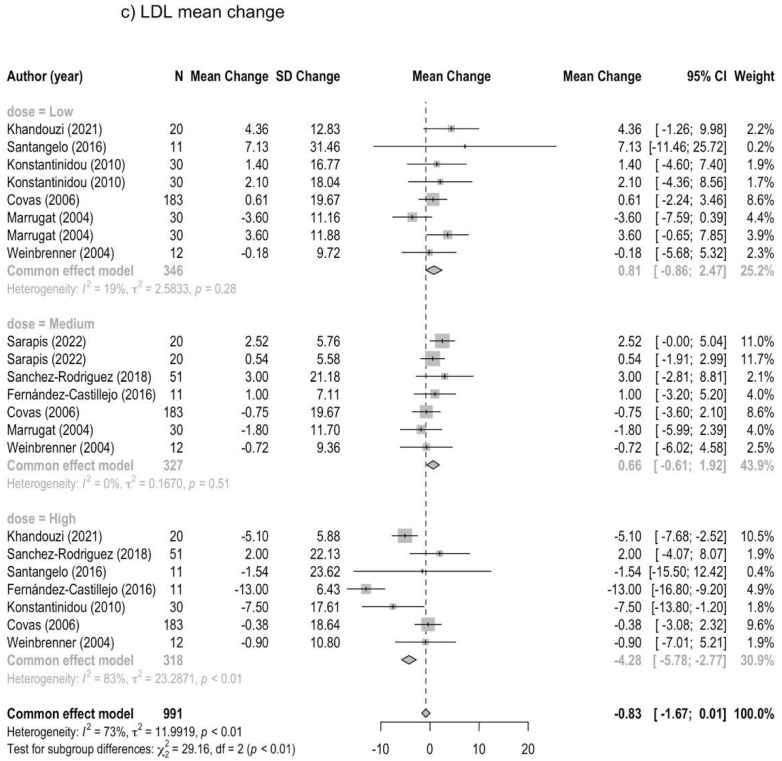
(**a**) Meta-analysis on HDL-C according to tertiles of daily exposure to olive oil polyphenols (low, medium, high). (**b**) Meta-analysis on serum total cholesterol according to tertiles of daily exposure to olive oil polyphenols (low, medium, high). (**c**) Meta-analysis on LDL-C according to tertiles of daily exposure to olive oil polyphenols (low, medium, high).

Meta-analyses on lipid markers (LDL-C, TC, and HDL-C) were structured by clustering the studies according to tertiles of daily exposure to olive oil polyphenols (low, medium, and high) (Figure 2a–c). When TC was recorded as the outcome, daily olive oil polyphenol consumption had no significant effect on TC levels in a total sample of 969 (WMD common effect model 0.49, 95%CI −0.55 to 1.53, heterogeneity 14%, *p* = 0.28), and similarly, WMDs on each level of exposure showed no effects for low (WMD 1.42, 95%CI −0.34 to 3.18), medium (WMD −0.05, 95%CI −1.73 to 1.62), or high (WMD 0.05, 95%CI −1.98; to 2.08) daily consumptions of olive oil polyphenols.

When LDL-C was considered as the outcome, daily consumption of olive oil polyphenols slightly but significantly reduced LDL-C levels in a total sample of 991 people (WMDs common effect model −0.83, 95%CI −1.67 to 0.01, heterogeneity 73%, *p* < 0.01), and WMDs for each level of daily exposure showed a strong significant effect on reducing LDL-C levels, yet only in individuals participating in a daily high consumption of olive oil polyphenols (WMD −4.28, 95%CI −5.78 to −2.77).

Lastly, when HDL-C was the considered outcome, the daily consumption of olive oil polyphenols showed a statistically significant effect on enhancing HDL-C levels in a total sample of 991 subjects (WMD common effect model 1.13, 95%CI 0.45; 1.80, heterogeneity 38%, *p* = 0.04), and WMDs for each level of daily exposure consistently showed a statistically significant effect and improved HDL-C levels following low (WMD 0.66, 95%CI 0.10 to 1.23), medium (WMD 1.36, 95%CI 0.76; 1.95), and high (WMD 1.13, 95%CI 0.45; 1.80) daily consumptions of olive oil polyphenols.

**Figure 3:**
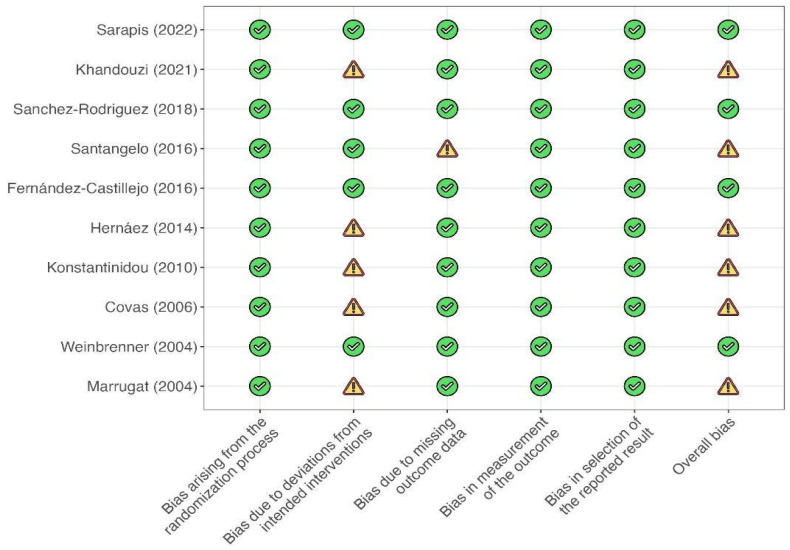
Risk of bias within selected studies.

**Figure 4:**
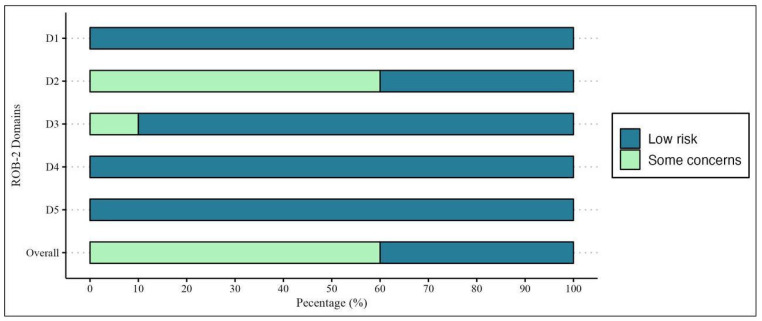
Risk of bias across selected studies.

The risk of bias was found to be low in 4 of the 10 RCTs. In contrast, some concerns were raised in others, mainly due to allocation concealment and the improper reporting of whether blinding was performed during the analysis (Figure 3 and Figure 4). Furthermore, one study [34] was found to be poor in domain three due to relevant loss at follow-up. 

## 4. Discussion

This systematic review with a meta-analysis of human RCT studies evaluated the relationship between the daily consumption of olive oil polyphenols and HDL-C, LDL-C, and TC serum concentrations, to assess the existence of a positive effect on HDL-C without causing other lipid markers to alter for the worse, which is a specific criterion required by the EFSA to substantiate a functional health claim. The basic assumption is that HDL-C acts as a cholesterol scavenger and is involved in the reverse transport of cholesterol in the body, from peripheral tissues to the liver. In contrast, LDL transports cholesterol from the liver to peripheral tissues, including the arteries. The maintenance of normal HDL-C—without increasing LDL-C levels—is recognized as a beneficial physiological effect by the EFSA, while a functional claim would be granted to a food or substance able to exert this effect.

Our findings showed that the daily consumption of olive oil polyphenols had no significant effect on TC levels in a total sample of 969 people (WMD pooled effect model 0.49, 95%CI −0.55 to 1.53, heterogeneity 14%, *p* = 0.28). However, when LDL-C was targeted, the daily consumption of olive oil polyphenols slightly but significantly reduced LDL-C levels in a total sample of 991 people (WMDs common effect model −0.83, 95%CI −1.67 to 0.01, heterogeneity 73%, *p* < 0.01), and the WMDs for each daily exposure level showed a strong significant effect on LDL-C reduction but only following a high daily consumption (268–600 mg/kg) of olive oil polyphenols (WMD −4.28, 95%CI −5.78 to −2.77). Interestingly, HDL-C levels were found to be significantly enhanced in a total sample of 991 subjects (WMD pooled effect model 1.13, 95%CI 0.45; 1.80, heterogeneity 38%, *p* = 0.04). The WMDs of each daily exposure level were also statistically significant, improving HDL-C levels following each level of consumption, i.e., low (WMD 0.66, 95%CI 0.10–1.23), medium (WMD 1.36, 95%CI 0.76–1.95), and high (WMD 1.13, 95%CI 0.45–1.80).

Currently, the beneficial role of olive oil consumption is widely recognized, whereby the EFSA has approved a health claim (Commission Regulation (EU) 432/2012) indicating that olive oil polyphenols contribute to the protection of blood lipids from oxidative stress [41]. However, the claim may only be used for olive oils that contain at least 5 mg of hydroxytyrosol and its derivatives (e.g., oleuropein complex and tyrosol) per 20 g of olive oil. Thus, to bear the claim, information shall be given to the consumer that the beneficial effect is obtained following a daily intake of 20 g of olive oil. Indeed, present evidence has currently demonstrated that phenolic compounds, particularly hydroxytyrosol and oleuropein, may dose-dependently inhibit LDL and HDL oxidation both in vitro and in vivo, while also repressing superoxide-driven reactions, and disrupting the chain propagation of lipid peroxides [42,43]. Interestingly, a 2006 multicenter report by Covas et al. demonstrated a linear increase in HDL cholesterol levels in 200 healthy males following the administration of olive oils containing low, medium, and high polyphenol contents for 3 weeks [38]. Furthermore, the TC/HDL-C ratio was found to decrease linearly with the phenolic content of the olive oil [38]. However, this latter report on its own failed to validate a claim on the protective effect of maintaining optimal HDL cholesterol values in 2012 [26]. 

This meta-analysis of 10 RCTs adds new evidence based on the exposure to olive oil, and specifically its polyphenols, whereby it significantly improves circulating HDL-C levels without affecting the TC levels, while also potentially reducing circulating LDL-C levels following the consumption of high daily concentrations (268–600 mg/kg). 

As for the biological path, it should be noted that the main phenols found in the olive plant and olive oil are oleuropein, tyrosol, and hydroxytyrosol. These share some potential to target various enzymes, and signaling molecules and to directly affect the transcription of various proteins, including lipid-binding, transport, or metabolizing enzymes [19,44,45,46,47]. Previous research has shown that oleuropein is a ligand of peroxisome proliferator-activated receptor-alpha (PPAR-α) [48]. PPAR-α agonists can effectively modulate lipid profiles, and these agonists are currently used as important targets for the treatment of dyslipidemia as well as insulin resistance [49]. In addition, PPAR-α activation favorably reduces the expression of proinflammatory genes and affects serum lipid levels. Further, oleuropein was shown to decrease the activity of hydroxymethylglutaryl-CoA reductase, which led to a reduction in cholesterol synthesis in rat hepatocytes [18,50]. Then, olive polyphenols might also affect bile flow and secondarily promote fecal excretion of lipids by increasing cholesterol and bile acid concentrations [51]. Previous evidence has shown that diet plays a major role in the balance of gut microbiota and bile acid homeostasis [52]. Specifically, population studies showing increased consumption of fruits and vegetables high in polyphenols have been associated with increased growth of probiotic bacteria that actively interact with bile acid metabolizing activity [53]. On this latter point, an additional interesting mechanism, which was recently studied, might be mediated by the intestinal bacterial flora. Indeed, although most phenols are rapidly absorbed in the small intestine, some simple and complex phenols continue to reach the large intestine, where they are metabolized by the intestinal microbiota. A bulk fermentation study of the human colonic microbiota revealed that probiotics, represented particularly by Bifidobacterium and Lactobacillus, absorb oleuropein as a carbon source in the proliferation process. Therefore, oleuropein, acting as an antioxidant, also has a prebiotic effect and is used as a carbon source during the proliferation phase by probiotics such as Bifidobacterium and Lactobacillus [54]. Probiotics can also modify the intestinal environment by affecting histone deacetylation, which in turn affects the permeability of the intestinal epithelium. This has a significant impact on reducing TC, LDL, and CVD risk factors. Probiotics protect intestinal barrier function, inhibit the production of proinflammatory factors (TNF-α, IL-6), promote mucin production, and reduce LDL and TC levels [55]. Bifidobacterium and Lactobacillus are frequently used in the treatment of hypertension, hyperlipidemia, and type 2 diabetes. In addition, probiotics can also be used to address cardiovascular risk factors [56].

Given these findings, it is worth pointing out that not all commercially available olive oils have the same polyphenol content. The lack of regulatory frameworks for labeling the concentration of polyphenols in food and olive oil limits consumer claims, while also minimizing the intrinsic potential of olive oil as a core Mediterranean health product. Further, it is difficult to predict daily intake of olive oil as it is consumed as a dressing rather than a food, and its antioxidant effect is variably impaired by exposure to cooking temperature [57]. However, this research provides strong evidence to support the benefits offered by olive oil polyphenols, and we are confident it may support food policies regarding transparency of product qualities and nutritional benefits to the consumer.

A potential limitation in our meta-analysis concerns the lack of differentiation between the olive oil cultivars used in the selected studies, making the monounsaturated fatty acid profile heterogeneous, and the use of the extra virgin variety, i.e., the juice of the ripe olive fruit obtained only by cold pressing [10,58,59]. The inability to differentiate olive oil types could be a potential explanation for the heterogeneity observed in some effect sizes. Another limitation lies in population heterogeneity, as 2 of the 10 RCTs were non-European-based, thus, implying the use of different baseline dietary patterns as well. In addition, there was slight heterogeneity concerning the study designs (there were a couple of parallel-arm RCTs) and the duration of the intervention, although a 3-week length prevailed. Finally, the sample power of studies was not always justified, with the presence of a minority of diabetics and hypercholesterolemic subjects, and the inconsistency of the washout period across studies posed other limitations to this meta-analysis.

## 5. Conclusions

The healthy benefits of olive oil extend further than its lipid fraction, as its polyphenols showed potential in maintaining lipid metabolism. Labeling frameworks should highlight this health feature of extra virgin olive oil as the main constituent of the Mediterranean diet, whereby a higher polyphenol content occurs from mechanical extraction. Moreover, it is also advisable to declare the polyphenol content of this product in the market. Therefore, consumers will be made more aware of the quality and possible health effects of the products they are consuming since the implementation of nutrition labels offers the best means of information.

## Figures and Tables

**Table 1:** Descriptions of selected RCT studies (*n* = 10).

Author, Year [Ref.]	Country	Population	Design	Inclusion Criteria	Exclusion Criteria	Washout Period	*n*	Age	Daily Dose (mL)	Intervention Duration
Sarapis, 2022 [31]	Australia	Healthy adults	Double-blind, cross-over RCT	BMI between 18.5–40 kg/m^2^	Smoking, pregnant, or lactating women, hyperlipidemia, diabetes, hypertension, inflammatory conditions (e.g., rheumatoid arthritis), intestinal disease (e.g., inflammatory bowel disease; irritable bowel syndrome), food intolerances, blood coagulation disorders, and any cognitive or mood disorder	2-weeks	20	20–70	60	3-weeks
Khandouzi, 2021 [32]	Iran	Men and post-menopausal women	Parallel-arm, RCT	Having at least one of the major cardiovascular risk factors including hypertension, diabetes mellitus, dyslipidemia, or acute cardiac events.	Administering anti-inflammatory medication, dietary antioxidants, or omega-3 supplements the month before the study, consumption of less than 80% of the olive oil delivered to the participants, lipid-lowering medications or coronary artery bypass graft (CABG), and gastrointestinal complications, such as diarrhea.	Not applicable	20	20–75	25	6-weeks
Sanchez-Rodriguez, 2018 [33]	Spain	General population	Double-blind, cross-over RCT	Being in good health based on a physical examination and basic biochemical and hematological analyses, and willingness to provide written informed consent. basic biochemical and hematological analyses,	Smoking, intake of antioxidant supplements, aspirin or any other drugs with established antioxidant properties, hyperlipidemia, obesity (BMI > 30 kg/m^2^), diabetes, hypertension, celiac, or other intestinal disease, any condition limiting mobility, and life-threatening diseases.	2-weeks	51	20–50	30	3-weeks
Santangelo, 2016 [34]	Italy	T2D patients	Cross-over RCT	Type 2 diabetes	Age > 80 years, smoking habits, use of antioxidant supplements, insulin treatment, use of aspirin or drugs with established antioxidant properties, history of CVD, any severe chronic illness, liver and renal failure, hormone replacement therapy, hyper- or hypothyroidism, drug or alcoholic addiction, severe history of allergy, or intolerance to olive oil	4-weeks	11	55–80	25	4-weeks
Fernández-Castillejo, 2016 [35]	Spain	Hypercholesterolemic (TC > 200 mg/dL)	Double blind, cross-over RCT	Normal blood pressure, hypercholesterolemic	Smoking, LDL-C > 190 mg/dL, TG > 350 mg/dL, fasting blood glucose > 126 mg/dL, plasma creatinine levels > 1.4 mg/dL for women and >1.5 mg/dL for men, BMI > 35, smokers (>1 cigarette/day), athletes with physical activity (>3000 METS min/day), hypertension, multiple allergies, intestinal diseases, chronic diseases (i.e., diabetes, cardiovascular)	2-weeks	11	40–70	25	3-weeks
Hernáez, 2014 [36]	Germany, Finland, Spain	Healthy male volunteers	Cross-over RCT	Healthy men	Smoking, use of antioxidant supplements, aspirin or any drug with antioxidant properties, hyperlipidemia, diabetes, intestinal disease	2-weeks	47	20–50	25	3-weeks
Konstantinidou, 2010 [37]	UK	Community	Parallel RCT	Healthy	Intake of antioxidant supplements, intake of acetosalicylic acid or any other drug with established antioxidative proper- ties, high levels of physical activity (3000 kcal/week in leisure time physical activity), obesity (body mass index (BMI) > 30 kg/m^2^), hypercholesterolemia (total cholesterol > 8.0 mM or dyslipidemia therapy), diabetes (glucose > 126 mg/dL or diabetes treatment); hypertension	Not applicable	30	20–50	25	3 months
Covas, 2006 [38]	Spain, Denmark, Germany, Italy, Finland	Healthy male volunteers	Cross-over RCT	Healthy men	Smoking, use of antioxidant supplements, aspirin or any drug with antioxidant properties, hyperlipidemia, diabetes, intestinal disease	2-weeks	183	20–60	25	3-weeks
Marrugat, 2004 [39]	Spain	Healthy male	Cross-over RCT	Non-smoking volunteers	Smoking, intake of antioxidant supplements, aspirin or any other drug with established antioxidant properties, obesity (body mass index > 30 kg/m^2^), dyslipidemia, diabetes, celiac or other intestinal disease, any condition limiting mobility, life-threatening diseases, or any other disease or condition that would impair compliance	2-weeks	30	20–70	25	4-weeks
Weinbrenner, 2004 [40]	Spain	Healthy male	Double blind, cross-over RCT	Healthy by medical history, a complete physical examination, and standard laboratory tests.	Intake of antioxidant supplements, aspirin or any other drug with established antioxidant properties, obesity (BMI 30 kg/m^2^), diabetes, hyperlipidemia, intestinal diseases, physical activity 12.6 MJ/w, or any condition that would impair compliance.	10 days	12	20–22	25	4 days

## Data Availability

No new data were created or analyzed in this study. Data sharing is not applicable to this article.
